# Joint between-sample normalization and differential expression detection through *ℓ*_0_-regularized regression

**DOI:** 10.1186/s12859-019-3070-4

**Published:** 2019-12-02

**Authors:** Kefei Liu, Li Shen, Hui Jiang

**Affiliations:** 1 Department of Biostatistics, Epidemiology and Informatics, University of Pennsylvania, 423 Guardian Dr, Philadelphia, PA, 19104 USA; 2 Department of Biostatistics, University of Michigan, 1415 Washington Heights, Ann Arbor, MI, 48109 USA

**Keywords:** Differential expression, Between-sample normalization, *ℓ*_0_-regularized regression, RNA-seq

## Abstract

**Background:**

A fundamental problem in RNA-seq data analysis is to identify genes or exons that are differentially expressed with varying experimental conditions based on the read counts. The relativeness of RNA-seq measurements makes the between-sample normalization of read counts an essential step in differential expression (DE) analysis. In most existing methods, the normalization step is performed prior to the DE analysis. Recently, Jiang and Zhan proposed a statistical method which introduces sample-specific normalization parameters into a joint model, which allows for simultaneous normalization and differential expression analysis from log-transformed RNA-seq data. Furthermore, an *ℓ*_0_ penalty is used to yield a sparse solution which selects a subset of DE genes. The experimental conditions are restricted to be categorical in their work.

**Results:**

In this paper, we generalize Jiang and Zhan’s method to handle experimental conditions that are measured in continuous variables. As a result, genes with expression levels associated with a single or multiple covariates can be detected. As the problem being high-dimensional, non-differentiable and non-convex, we develop an efficient algorithm for model fitting.

**Conclusions:**

Experiments on synthetic data demonstrate that the proposed method outperforms existing methods in terms of detection accuracy when a large fraction of genes are differentially expressed in an asymmetric manner, and the performance gain becomes more substantial for larger sample sizes. We also apply our method to a real prostate cancer RNA-seq dataset to identify genes associated with pre-operative prostate-specific antigen (PSA) levels in patients.

## Introduction

A fundamental problem in RNA-seq data analysis is to identify genes or exons that are differentially expressed with varying experimental conditions based on the read counts. Some widely used methods for differential expression analysis in RNA-seq data are edgeR [1, 2], DESeq2 [3] and limma-voom [4, 5]. In edgeR and DESeq2, the read counts are assumed to follow negative binomial (NB) distributions; while in limma-voom, the logarithmic transformation is taken on the data which compresses the dynamic range of the read counts so that the outliers become more “normal”. Consequently, existing statistical methods that are designed for analyzing normally distributed data can be employed to analyze RNA-seq data.

Due to the relative nature of RNA-seq measurements for transcript abundances as well as differences in library sizes and sequencing depths across samples [6], between-sample normalization of read counts is essential in differential expression (DE) analysis with RNA-seq data. A widely used approach for data normalization in RNA-seq is to employ a sample-specific scaling factor, e.g., CPM/RPM (counts/reads per million) [7], upper-quartile normalization [8], trimmed mean of M values [7] and DESeq normalization [9]. A review of normalization methods in RNA-data data analysis is given in [6]. In most existing methods for DE analysis in RNA-seq, the normalization step is performed prior to the DE detection step, which is sub-optimal because ideally normalization should be based on non-DE genes for which the complete list is unknown until after the DE analysis.

In [10], a statistical method for robust DE analysis using log-transformed RNA-seq data is proposed, where sample-specific normalization factors are introduced as unknown parameters. This allows for more accurate and reliable detection of DE genes by simultaneously performing between-sample normalization and DE detection. An *ℓ*_0_ penalty is introduced to enforce that a subset of genes are selected as being differentially expressed. The experimental conditions are restricted to be categorical (e.g., 0 and 1 for control and treatment groups, respectively), and a one-way analysis of variance (ANOVA) type model is employed to detect differentially expressed genes across two or more experimental conditions.

In [11], the model of [10] is generalized to continuous experimental conditions, and the sparsity-inducing *ℓ*_0_ penalty is relaxed as the *ℓ*_1_ penalty. An alternating direction method of multipliers (ADMM) algorithm is developed to solve the resultant convex problem. Due to the relaxation of the *ℓ*_0_ regularization, the method in [11] may not be as robust against noise and efficient in inducing sparse solutions as that in [10]. In this paper, we again generalize the model in [10] from categorical to continuous experimental conditions. But different from [11], we retain the *ℓ*_0_ penalty in our model to efficiently induce sparsity. We formulated two hypothesis tests suited to different applications: the first hypothesis test is that considered in [10] and answers the question of whether the expression of a gene is significantly affected by any covariate; and in addition, a second hypothesis is formulated to test whether the expression of a gene is significantly affected by a particular covariate, when all other covariates in the regression model are adjusted for.

Due to the use of the *ℓ*_0_ penalty, the resulting problem is high-dimensional, non-differentiable and non-convex. To fit the proposed model, we study the optimality conditions of the problem and develop an efficient algorithm for its solution. We also propose a simple rule for the selection of tuning parameters. Experiments on synthetic data demonstrate that the proposed method outperforms existing ones in terms of detection accuracy when a large fraction of genes are differentially expressed in an asymmetric manner, and the performance gain becomes more substantial for larger sample sizes. We also apply our method to a real prostate cancer RNA-seq dataset to identify genes associated with pre-operative prostate-specific antigen (PSA) levels in patients.

## Methods

Given *m* genes and *n* samples, let *y*_*ij*_,*i*=1,…,*m,j*=1,…,*n*, be the log-transformed gene expression values of the *i*-th gene in the *j*-th sample. A small positive constant can be added prior to taking the logarithm to avoid taking logarithm of zeros. We formulate the following model:

1 $$ y_{ij} = \alpha_{i}+\boldsymbol{\beta}_{i}^{\mathrm{T}} \boldsymbol{x}_{j} + d_{j} + \varepsilon_{ij},  $$

where *α*_*i*_ is the intercept,

2 $$ \boldsymbol{\beta}_{i} = \left[\begin{array}{c} \beta_{i1} \\ \beta_{i2} \\ \vdots \\ \beta_{ip} \\ \end{array}\right]\in \mathbb{R}^{p \times 1}  $$

is the regression coefficient vector of the linear model for gene *i*, and

3 $$ \boldsymbol{x}_{j} = \left[\begin{array}{c} x_{j1} \\ x_{j2} \\ \vdots \\ x_{jp} \\ \end{array}\right] \in \mathbb{R}^{p \times 1}  $$

is a vector of *p* predictor variables for sample *j* representing its experimental conditions (drug dosage, blood pressure, age, BMI, etc.), *d*_*j*_ represents the normalization factor for sample *j*, and $\varepsilon _{ij} \sim \mathcal {N}\left (0,\sigma _{i}^{2}\right)$ is i.i.d. Gaussian noise. Our goal is for each gene to determine whether its expression level is significantly associated with the experimental conditions or not.

### **Remark 1**

The *α*_*i*_ and *d*_*j*_ in (1) model gene-specific factors (e.g., gene length) and sample-specific factors (i.e., sequencing depth), respectively. Thus, model (1) can accommodate any gene expression levels summarized in the form of *c*_*ij*_/(*l*_*i*_·*q*_*j*_), where *c*_*ij*_ is the read count, *l*_*i*_ is the gene-specific scaling factor (e.g., gene length) associated with gene *i* and *q*_*j*_ is the sample-specific scaling factor (e.g., sequencing depth) associated with sample *j*. Special cases are read count (i.e., *l*_*i*_=*q*_*j*_=1), CPM/RPM (i.e., *l*_*i*_=1) *[*7*]*, RPKM/FPKM *[*12*,*^13^*]* and TPM *[*14*]*.

Since the random noise in gene expression measurements are independent across genes and samples, the likelihood is given by

4 $$ {\begin{aligned} &L\left(\boldsymbol{\alpha}, \{\boldsymbol{\beta}_{i}\}_{i=1}^{m}, \{\sigma^{2}_{i}\}_{i=1}^{m}, \boldsymbol{d}; \boldsymbol{Y} \right) \\&= {\prod_{i=1}^{m}} {\prod_{j=1}^{n}} \frac{1}{\sqrt{2\pi \sigma_{i}^{2}}} \exp\left\{ -\frac{\left(y_{ij}-\alpha_{i}-\boldsymbol{\beta}_{i}^{\mathrm{T}} \boldsymbol{x}_{j}-d_{j}\right)^{2}}{2\sigma_{i}^{2}} \right\}. \end{aligned}}  $$

The negative log-likelihood is

5 $$ {}{\begin{aligned} l\left(\boldsymbol{\alpha}, \{\boldsymbol{\beta}_{i}\}_{i=1}^{m}, \{\sigma^{2}_{i}\}_{i=1}^{m}, \boldsymbol{d}; \boldsymbol{Y} \right) = {\sum_{i=1}^{m}\sum_{j=1}^{n}} \frac{1}{2\sigma_{i}^{2}} \left(y_{ij}-\alpha_{i}-\boldsymbol{\beta}_{i}^{\mathrm{T}} \boldsymbol{x}_{j}-d_{j}\right)^{2} + C, \end{aligned}}  $$

where *C* depends on $\left \{\sigma _{i}^{2}\right \}$ but not on {*α*_*i*_},{***β***_*i*_} and {*d*_*j*_}. In “Maximum likelihood estimation of noise variance” section, we will describe how to estimate $\sigma _{i}^{2}, i=1,\dots,m$. Hereafter, we assume that $\sigma _{i}^{2}$’s are known and simply denote the negative log-likelihood as $l\left (\boldsymbol {\alpha }, \{\boldsymbol {\beta }_{i}\}_{i=1}^{m}, \boldsymbol {d}; \boldsymbol {Y} \right)$.

In practice, typically only a subset of genes are differentially expressed. We introduce a sparse penalty to the negative log likelihood function:

6 $$ {}{\begin{aligned} \text{min} \; f\left(\boldsymbol{\alpha}, \{\boldsymbol{\beta}_{i}\}_{i=1}^{m}, \boldsymbol{d}\right) &= {\sum_{i=1}^{m}\sum_{j=1}^{n}} \frac{1}{2\sigma_{i}^{2}} \left(y_{ij}-\alpha_{i}-\boldsymbol{x}_{j}^{\mathrm{T}} \boldsymbol{\beta}_{i}-d_{j}\right)^{2}\\ &\quad+ {\sum_{i=1}^{m}} \lambda_{i} \, p\left(\boldsymbol{\beta}_{i}\right), \end{aligned}}  $$

where *λ*_*i*_’s are tuning parameters that control the sparsity level of the solution, and *p*(***β***_*i*_) is a penalty function

In this paper, we use the following two types of penalty functions.

i) Type I penalty:
  7 $$ p\left(\boldsymbol{\beta}_{i}\right) = 1_{\boldsymbol{\beta}_{i} \neq \boldsymbol{0}}.  $$
  This penalty function applies to applications where all covariates are of interest and we want to identify genes for which at least one covariate is associated with its expression.
ii) Type II penalty:
  8 $$ p\left(\boldsymbol{\beta}_{i}\right) = 1_{\beta_{ip} \neq 0}.  $$
  This penalty applies to applications where only one (the *p*-th) covariate is of main interest (e.g., treatment) while we want to adjust for all other covariates (e.g., age, sex, etc).

### Algorithm development

Note that without *d*_*j*_, model (1) would be decoupled as *m* independent linear regression models, one for each gene. The first step of our algorithm is to solve for *d*_*j*_ and express it as a function of ***β***_*i*_’s.

Note that the optimization problem (6) is convex in (***α***,***d***). Therefore, the minimizer of (***α***,***d***) is one of its stationary points.

Taking partial derivatives of $f\left (\boldsymbol {\alpha }, \{\boldsymbol {\beta }_{i}\}_{i=1}^{m}, \boldsymbol {d}\right)$ with respect to *d*_*j*_,*j*=1,…,*n*, and setting them to zeros, we have

9 $$ d_{j} = \frac{1}{{\sum_{i=1}^{m}} \frac{1}{\sigma_{i}^{2}}} {\sum\limits_{i=1}^{m}} \frac{1}{\sigma_{i}^{2}} \left(y_{ij}-\alpha_{i}-\boldsymbol{x}_{j}^{\mathrm{T}} \boldsymbol{\beta}_{i}\right).  $$

The solution to model (1) is not unique because an arbitrary constant can be added to *d*_*j*_’s and subtracted from *α*_*i*_’s, while having the same model fit. To address this issue, we fix *d*_1_=0. Therefore

10 $$ d_{j} = d_{j} -d_{1} = \left(\bar{y}_{\cdot j}^{(w)}-\bar{y}_{\cdot 1}^{(w)}\right) - \left(\boldsymbol{x}_{j}-\boldsymbol{x}_{1}\right)^{\mathrm{T}} \boldsymbol{\bar{\beta}}^{(w)},  $$

where

11 $$ \bar{y}_{\cdot j}^{(w)} := \frac{1}{{\sum\limits_{i=1}^{m}} \frac{1}{\sigma_{i}^{2}}} {\sum_{i=1}^{m}} \frac{1}{\sigma_{i}^{2}} y_{ij}, \ \text{ for} \ j=1,\dots,n,  $$

12 $$ \boldsymbol{\bar{\beta}}^{(w)} := \frac{1}{{\sum\limits_{i=1}^{m}} \frac{1}{\sigma_{i}^{2}}} {\sum_{i=1}^{m}} \frac{1}{\sigma_{i}^{2}} \boldsymbol{\beta}_{i}.  $$

Here the superscript ^(*w*)^ denotes “weighted mean".

Calculating the partial derivatives of $f\left (\boldsymbol {\alpha }, \{\boldsymbol {\beta }_{i}\}_{i=1}^{m}, \boldsymbol {d}\right)$ with respect to *α*_*i*_,*i*=1,…,*m*, and setting them to zeros, we have

13 $$ \alpha_{i} = \frac{1}{n} {\sum_{j=1}^{n}} \left(y_{ij}-\boldsymbol{x}_{j}^{\mathrm{T}} \boldsymbol{\beta}_{i}-d_{j}\right) = \bar{y}_{i\cdot} - \boldsymbol{\bar{x}}^{\mathrm{T}} \boldsymbol{\beta}_{i} - \frac{1}{n} {\sum_{j=1}^{n}} d_{j},  $$

where

14 $$ \bar{y}_{i\cdot} := \frac{1}{n} {\sum_{j=1}^{n}} y_{ij}, \; i=1,\dots,m  $$

15 $$ \boldsymbol{\bar{x}} := \frac{1}{n} {\sum_{j=1}^{n}} \boldsymbol{x}_{j}.  $$

From (10) it follows

16 $$ \frac{1}{n} {\sum_{j=1}^{n}} d_{j} = \left(\bar{y}^{(w)} - \bar{y}_{\cdot 1}^{(w)}\right) - \left(\boldsymbol{\bar{x}}-\boldsymbol{x}_{1}\right)^{\mathrm{T}} \boldsymbol{\bar{\beta}}^{(w)},  $$

where

17 $$ \bar{y}^{(w)} := \frac{1}{{\sum\limits_{i=1}^{m}} \frac{1}{\sigma_{i}^{2}}} {\sum_{i=1}^{m}} \frac{1}{\sigma_{i}^{2}} \cdot \frac{1}{n} {\sum_{j=1}^{n}} y_{ij}.  $$

Substituting (16) into (13) yields

18 $$ \alpha_{i} = \bar{y}_{i\cdot} + \bar{y}_{\cdot 1}^{(w)} - \bar{y}^{(w)} + \left(\boldsymbol{\bar{x}}-\boldsymbol{x}_{1}\right)^{\mathrm{T}} \boldsymbol{\bar{\beta}}^{(w)} - \boldsymbol{\bar{x}}^{\mathrm{T}} \boldsymbol{\beta}_{i}.  $$

The sum of (10) and (18) yields

19 $$ \alpha_{i} + d_{j} = \bar{y}_{i\cdot} + \bar{y}_{\cdot j}^{(w)} - \bar{y}^{(w)} -\left(\boldsymbol{x}_{j}-\boldsymbol{\bar{x}}\right)^{\mathrm{T}} \boldsymbol{\bar{\beta}}^{(w)} - \boldsymbol{\bar{x}}^{\mathrm{T}} \boldsymbol{\beta}_{i}.  $$

Substituting (19) into (6), the problem becomes an *ℓ*_0_-regularized linear regression problem with $\{\boldsymbol {\beta }_{i}\}_{i=1}^{m}$ being the only variables to be optimized:

20 $$ {}{\begin{aligned} \underset{\{\boldsymbol{\beta}_{i}\}_{i=1}^{m}}{\text{minimize}} \; f\left(\{\boldsymbol{\beta}_{i}\}_{i=1}^{m}\right) &= {\sum_{i=1}^{m}} \frac{1}{2\sigma_{i}^{2}} {\sum_{j=1}^{n}} \left(\tilde{y}_{ij} + \boldsymbol{\tilde{x}}_{j}^{\mathrm{T}} \boldsymbol{\bar{\beta}}^{(w)} - \boldsymbol{\tilde{x}}_{j}^{\mathrm{T}} \boldsymbol{\beta}_{i}\right)^{2}\\ &\quad+ {\sum_{i=1}^{m}} \lambda_{i} \, p\left(\boldsymbol{\beta}_{i}\right), \end{aligned}}  $$

where

21 $$ \tilde{y}_{ij} := y_{ij} - \bar{y}_{i\cdot} - \bar{y}_{\cdot j}^{(w)} + \bar{y}^{(w)}  $$

22 $$ \boldsymbol{\tilde{x}}_{j} := \boldsymbol{x}_{j}-\boldsymbol{\bar{x}}.  $$

It is easy to see that

23 $$ {\sum_{i=1}^{m}} \frac{1}{\sigma_{i}^{2}} \tilde{y}_{ij} = 0, \quad {\sum_{j=1}^{n}} \tilde{y}_{ij} = 0.  $$

In the next two sections, we will describe algorithms to solve Problem (20) with type I and type II penalties, respectively.

#### Fitting the model with type I penalty

Denote $\boldsymbol {\delta }=\boldsymbol {\bar {\beta }}^{(w)}$, and let

24 $$ g_{i}\left(\boldsymbol{\beta}_{i}\right) = \frac{1}{2\sigma_{i}^{2}} {\sum_{j=1}^{n}} \left(\tilde{y}_{ij} + \boldsymbol{\tilde{x}}_{j}^{\mathrm{T}} \boldsymbol{\delta} - \boldsymbol{\tilde{x}}_{j}^{\mathrm{T}} \boldsymbol{\beta}_{i}\right)^{2} + \lambda_{i} 1_{\boldsymbol{\beta}_{i} \neq \boldsymbol{0}},  $$

where ***β***_*i*_’s are considered as functions of ***δ***. The objective in Problem (20) can be written as $f\left (\boldsymbol {\beta }\right) = {\sum _{i=1}^{m}} g_{i}\left (\boldsymbol {\beta }_{i}\right)$. Assume that ***δ*** is fixed, *f* can be minimized by minimizing each *g*_*i*_(***β***_*i*_) separately.

Next we express the minimizing solution of *g*_*i*_(***β***_*i*_) as a function of ***δ***.

When ***β***_*i*_=***0***,

25 $$ g_{i}\left(\boldsymbol{0}\right) = \frac{1}{2\sigma_{i}^{2}} {\sum_{j=1}^{n}} \left(\tilde{y}_{ij} + \boldsymbol{\tilde{x}}_{j}^{\mathrm{T}} \boldsymbol{\delta}\right)^{2}.  $$

When ***β***_*i*_≠***0***,

26 $$ g_{i}\left(\boldsymbol{\beta}_{i}\right) = \frac{1}{2\sigma_{i}^{2}} {\sum_{j=1}^{n}} \left(\tilde{y}_{ij} + \boldsymbol{\tilde{x}}_{j}^{\mathrm{T}} \boldsymbol{\delta} - \boldsymbol{\tilde{x}}_{j}^{\mathrm{T}} \boldsymbol{\beta}_{i}\right)^{2} + \lambda_{i}.  $$

Taking partial derivatives of (26) with respect to ***β***_*i*_,*i*=1,…,*m*, and setting them to zeros yields

27 $$ \boldsymbol{\beta}_{i}^{\text{(ols)}} = \left(\boldsymbol{\tilde{X}}^{\mathrm{T}} \boldsymbol{\tilde{X}}\right)^{-1} \boldsymbol{\tilde{X}}^{\mathrm{T}} \boldsymbol{\tilde{y}}_{i} + \boldsymbol{\delta},  $$

where the superscript ^(ols)^ indicates an ordinary least squares estimate for the model,

28 $$ \boldsymbol{\tilde{X}} = \left[\begin{array}{c} \boldsymbol{\tilde{x}}_{1}^{\mathrm{T}} \\ \boldsymbol{\tilde{x}}_{2}^{\mathrm{T}} \\ \vdots \\ \boldsymbol{\tilde{x}}_{n}^{\mathrm{T}} \\ \end{array}\right] = \left[\begin{array}{cccc} \tilde{x}_{11} & \tilde{x}_{12} & \cdots & \tilde{x}_{1p} \\ \tilde{x}_{21} & \tilde{x}_{22} & \cdots & \tilde{x}_{2p} \\ \vdots & \vdots & \ddots & \vdots \\ \tilde{x}_{n1} & \tilde{x}_{n2} & \cdots & \tilde{x}_{np} \\ \end{array}\right] \in \mathbb{R}^{n \times p},  $$

and $\boldsymbol {\tilde {y}}_{i}$ is a column vector containing the centered expression of gene *i* in all samples, i.e., the *i*-th row of $\boldsymbol {\tilde {Y}}$:

29 $$ \boldsymbol{\tilde{Y}} = \left[\begin{array}{c} \boldsymbol{\tilde{y}}_{1}^{\mathrm{T}} \\ \boldsymbol{\tilde{y}}_{2}^{\mathrm{T}} \\ \vdots \\ \boldsymbol{\tilde{y}}_{m}^{\mathrm{T}} \\ \end{array}\right] = \left[\begin{array}{cccc} \tilde{y}_{11} & \tilde{y}_{12} & \cdots & \tilde{y}_{1n} \\ \tilde{y}_{21} & \tilde{y}_{22} & \cdots & \tilde{y}_{2n} \\ \vdots & \vdots & \ddots & \vdots \\ \tilde{y}_{m1} & \tilde{y}_{m2} & \cdots & \tilde{y}_{mn} \\ \end{array}\right] \in \mathbb{R}^{m \times n}.  $$

The objective function value at $\boldsymbol {\beta }_{i}=\boldsymbol {\beta }_{i}^{\text {(ols)}}$ is

30 $$ g_{i}\left(\boldsymbol{\beta}_{i}^{\text{(ols)}}\right) = \frac{1}{2\sigma_{i}^{2}} \boldsymbol{\tilde{y}}_{i}^{\mathrm{T}} \left[ \boldsymbol{I}_{n} - \boldsymbol{\tilde{X}} \left(\boldsymbol{\tilde{X}}^{\mathrm{T}} \boldsymbol{\tilde{X}}\right)^{-1} \boldsymbol{\tilde{X}}^{\mathrm{T}} \right] \boldsymbol{\tilde{y}}_{i} + \lambda_{i}.  $$

The change in the objective value *g*_*i*_(***β***_*i*_) from $\boldsymbol {\beta }_{i}=\boldsymbol {\beta }_{i}^{\text {(ols)}} \neq 0$ in Eq. (30) to ***β***_*i*_=***0*** in Eq. (25) is

31 $$ {}{\begin{aligned} g_{i}\left(\boldsymbol{0}\right) - g_{i}\left(\boldsymbol{\beta}_{i}^{\text{(ols)}}\right) &=\; \frac{1}{2\sigma_{i}^{2}} \left[ \boldsymbol{\tilde{y}}_{i}^{\mathrm{T}} \boldsymbol{\tilde{X}} \left(\boldsymbol{\tilde{X}}^{\mathrm{T}} \boldsymbol{\tilde{X}}\right)^{-1} \boldsymbol{\tilde{X}}^{\mathrm{T}} \boldsymbol{\tilde{y}}_{i} + 2 \boldsymbol{\tilde{y}}_{i}^{\mathrm{T}} \boldsymbol{\tilde{X}} \boldsymbol{\delta} + \boldsymbol{\delta}^{\mathrm{T}} \boldsymbol{\tilde{X}}^{\mathrm{T}} \boldsymbol{\tilde{X}} \boldsymbol{\delta} \right] - \lambda_{i} \\ &=\; \frac{1}{2\sigma_{i}^{2}} \left\|\boldsymbol{\tilde{X}} \left[\left(\boldsymbol{\tilde{X}}^{\mathrm{T}} \boldsymbol{\tilde{X}}\right)^{-1} \boldsymbol{\tilde{X}}^{\mathrm{T}} \boldsymbol{\tilde{y}}_{i} + \boldsymbol{\delta}\right]\right\|^{2} - \lambda_{i}. \end{aligned}}  $$

Therefore, the solution is

32 $$ \boldsymbol{\beta}_{i} = \left\{ \begin{array}{ll} 0 &\text{ if} \frac{1}{2\sigma_{i}^{2}} \left\|\boldsymbol{\tilde{X}} \boldsymbol{\beta}_{i}^{\text{(ols)}}\right\|^{2} < \lambda_{i} \\ \boldsymbol{\beta}_{i}^{\text{(ols)}} &\text{ otherwise} \end{array}\right.  $$

Now we only need to solve for ***δ***. We have

33 $$ {}{\begin{aligned} \boldsymbol{\hat{\delta}} &= \arg \,\min_{\boldsymbol{\delta}} \sum_{i=1}^{m} \min \left\{g_{i}\left(\boldsymbol{0}\right), g_{i}\left(\boldsymbol{\beta}_{i}^{\text{(ols)}}\right)\right\} \\ &= \arg \,\min_{\boldsymbol{\delta}} \sum_{i=1}^{m} \min \left\{g_{i}\left(\boldsymbol{0}\right) - g_{i}\left(\boldsymbol{\beta}_{i}^{\text{(ols)}}\right), 0\right\} \\ &\small{=\arg \,\min_{\boldsymbol{\delta}} \sum_{i=1}^{m} \min \left\{\frac{1}{2\sigma_{i}^{2}} \left\|\boldsymbol{\tilde{X}} \left[\left(\boldsymbol{\tilde{X}}^{\mathrm{T}} \boldsymbol{\tilde{X}}\right)^{-1} \boldsymbol{\tilde{X}}^{\mathrm{T}} \boldsymbol{\tilde{y}}_{i} + \boldsymbol{\delta}\right]\right\|^{2} - \lambda_{i}, 0\right\}}\\ &\small= \arg \,\min_{\boldsymbol{\delta}} \sum_{i=1}^{m} \min \left\{\frac{1}{2\sigma_{i}^{2}} \left\|\boldsymbol{\tilde{X}} \left[\left(\boldsymbol{\tilde{X}}^{\mathrm{T}} \boldsymbol{\tilde{X}}\right)^{-1} \boldsymbol{\tilde{X}}^{\mathrm{T}} \boldsymbol{\tilde{y}}_{i} + \boldsymbol{\delta}\right]\right\|^{2}, \lambda_{i}\right\} \end{aligned}}  $$

where the second equality is due to the fact that $g_{i}\left (\boldsymbol {\beta }_{i}^{\text {(ols)}}\right)$ is a constant independent of ***δ***, and the third equality follows from (31). Problem (33) can be solved exactly using an exhausted grid search for *p*=1 or 2, and approximately using a general global optimization algorithm (e.g., the optim function in R) for larger *p*. A more efficient algorithm proposed in [15] can also be used.

After we obtain the estimate of ***δ***, we substitute it into (32) to get the estimate of ***β***_*i*_. Algorithm 1 describes the complete model fitting procedure. 
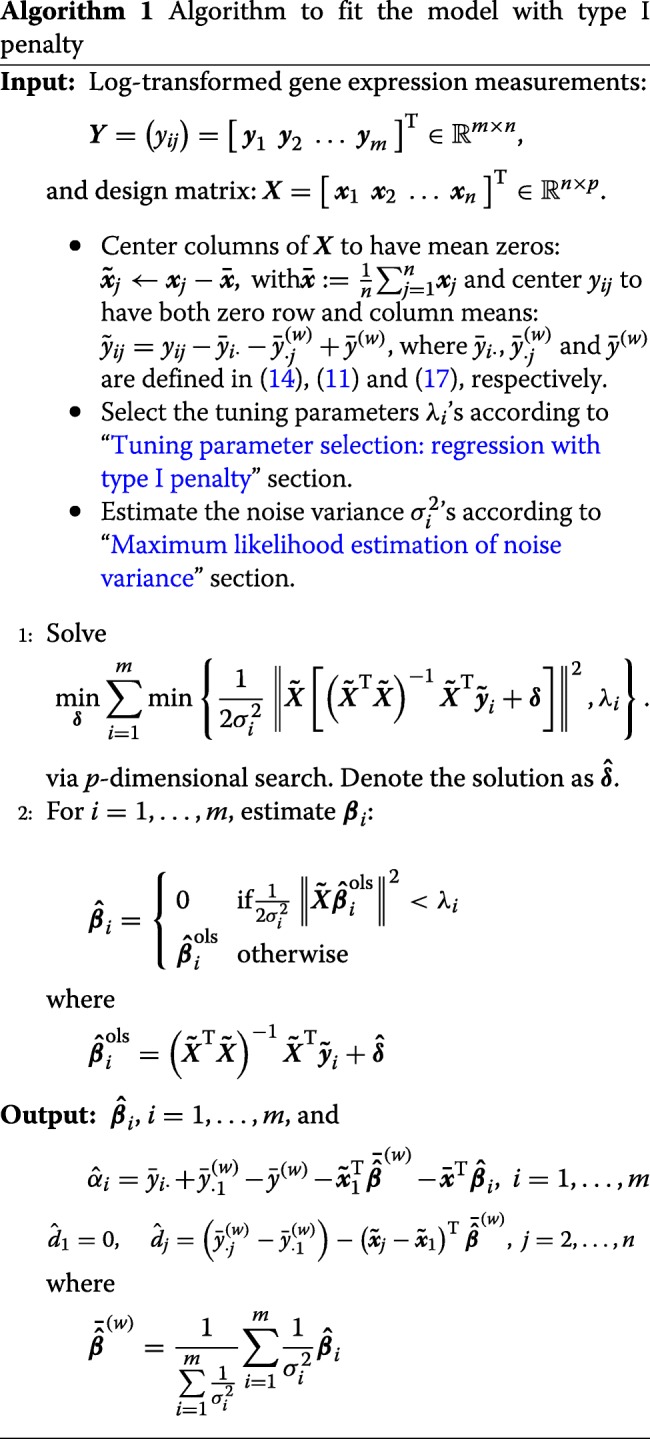


#### Fitting the model with type II penalty

Denote $\boldsymbol {\delta }=\boldsymbol {\bar {\beta }}^{(w)}$, and let

34 $$ h_{i}\left(\boldsymbol{\beta}_{i}\right) = \frac{1}{2\sigma_{i}^{2}} {\sum_{j=1}^{n}} \left(\tilde{y}_{ij} + \boldsymbol{\tilde{x}}_{j}^{\mathrm{T}} \boldsymbol{\delta} - \boldsymbol{\tilde{x}}_{j}^{\mathrm{T}} \boldsymbol{\beta}_{i}\right)^{2} + \lambda_{i} 1_{\beta_{ip} \neq 0},  $$

where ***β***_*i*_’s are considered as functions of ***δ***. The objective function in Eq. (20) is $f\left (\boldsymbol {\beta }\right) \,=\, {\sum \limits _{i=1}^{m}} h_{i}\left (\boldsymbol {\beta }_{i}\right)$. Assume that ***δ*** is fixed, *f* can be optimized by minimizing each *h*_*i*_(***β***_*i*_) separately.

Next we find the solution for ***β***_*i*_’s as a function of ***δ*** by minimizing *h*_*i*_(***β***_*i*_).

Denote

$$ {}{\begin{aligned} \boldsymbol{\tilde{x}}_{j} = \left[\begin{array}{c} \tilde{x}_{j1} \\ \tilde{x}_{j2} \\ \vdots \\ \tilde{x}_{jp} \\ \end{array}\right] = \left[\begin{array}{c} \boldsymbol{\tilde{x}}_{j}^{-} \\ \tilde{x}_{jp} \\ \end{array}\right], \boldsymbol{\beta}_{i} = \left[\begin{array}{c} \beta_{i1} \\ \beta_{i2} \\ \vdots \\ \beta_{ip} \\ \end{array}\right] = \left[\begin{array}{c} \boldsymbol{\beta}_{i}^{-} \\ \beta_{ip} \\ \end{array}\right], \boldsymbol{\delta} = \left[\begin{array}{c} \delta_{1} \\ \delta_{2} \\ \vdots \\ \delta_{p} \\ \end{array}\right] = \left[\begin{array}{c} \boldsymbol{\delta}^{-} \\ \delta_{p} \\ \end{array}\right]. \end{aligned}} $$

When *β*_*ip*_=0,

35 $$ h_{i}\left(\boldsymbol{\beta}_{i}\right) = \frac{1}{2\sigma_{i}^{2}} {\sum_{j=1}^{n}} \left(\tilde{y}_{ij} + \boldsymbol{\tilde{x}}_{j}^{\mathrm{T}} \boldsymbol{\delta} - {\boldsymbol{\tilde{x}}_{j}^{-}}^{\mathrm{T}} \boldsymbol{\beta}_{i}^{-}\right)^{2}.  $$

Taking derivatives of (35) with respect to $\boldsymbol {\beta }_{i}^{-}, i=1,\ldots,m,$ and setting them to zeros yields

36 $$ \boldsymbol{\beta}_{i}^{-} = \left({\boldsymbol{\tilde{X}}^{-}}^{\mathrm{T}} {\boldsymbol{\tilde{X}}^{-}}\right)^{-1} {\boldsymbol{\tilde{X}}^{-}}^{\mathrm{T}} \left(\boldsymbol{\tilde{y}}_{i} + \boldsymbol{\tilde{x}}^{p} \delta_{p} \right) + \boldsymbol{\delta}^{-},  $$

where

37 $$ {}{\begin{aligned} \boldsymbol{\tilde{X}}^{-} =\! \left[\begin{array}{c} {\boldsymbol{\tilde{x}}_{1}^{-}}^{\mathrm{T}} \\ {\boldsymbol{\tilde{x}}_{2}^{-}}^{\mathrm{T}} \\ \vdots \\ {\boldsymbol{\tilde{x}}_{n}^{-}}^{\mathrm{T}} \\ \end{array}\right] = \left[\begin{array}{cccc} \tilde{x}_{11} & \tilde{x}_{12} & \cdots & \tilde{x}_{1,p-1} \\ \tilde{x}_{21} & \tilde{x}_{22} & \cdots & \tilde{x}_{2,p-1} \\ \vdots & \vdots & \ddots & \vdots \\ \tilde{x}_{n1} & \tilde{x}_{n2} & \cdots & \tilde{x}_{n,p-1} \\ \end{array}\right] \in \mathbb{R}^{n \times (p-1)}, \quad \boldsymbol{\tilde{x}}^{p} = \left[\begin{array}{c} \tilde{x}_{1p} \\ \tilde{x}_{2p} \\ \vdots \\ \tilde{x}_{np} \\ \end{array}\right]. \end{aligned}}  $$

Denote $\boldsymbol {\beta }_{i}^{\text {(r)}} = \left [\begin {array}{c} \boldsymbol {\beta }_{i}^{-} \\ 0 \\ \end {array}\right ]$, where the superscript ^(r)^ denotes the reduced model. Substituting $\boldsymbol {\beta }_{i} = \boldsymbol {\beta }_{i}^{\text {(r)}}$ into (35) and after some matrix algebraic manipulation, we have

38 $$ {}{\begin{aligned} h_{i}\left(\boldsymbol{\beta}_{i}^{\text{(r)}}\right) = \frac{1}{2\sigma_{i}^{2}} \left(\boldsymbol{\tilde{y}}_{i} + \boldsymbol{\tilde{x}}^{p} \delta_{p} \right)^{\mathrm{T}} \left[ \boldsymbol{I}_{n} - {\boldsymbol{\tilde{X}}^{-}} \left({\boldsymbol{\tilde{X}}^{-}}^{\mathrm{T}} {\boldsymbol{\tilde{X}}^{-}}\right)^{-1} {\boldsymbol{\tilde{X}}^{-}}^{\mathrm{T}} \right] \left(\boldsymbol{\tilde{y}}_{i} + \boldsymbol{\tilde{x}}^{p} \delta_{p} \right). \end{aligned}}  $$

When *β*_*ip*_≠0,

39 $$ h_{i}\left(\boldsymbol{\beta}_{i}\right) = \frac{1}{2\sigma_{i}^{2}} {\sum_{j=1}^{n}} \left(\tilde{y}_{ij} + \boldsymbol{\tilde{x}}_{j}^{\mathrm{T}} \boldsymbol{\delta} - \boldsymbol{\tilde{x}}_{j}^{\mathrm{T}} \boldsymbol{\beta}_{i}\right)^{2} + \lambda_{i}.  $$

The minimizing solution of *h*_*i*_(***β***_*i*_) is $\boldsymbol {\beta }_{i}^{\text {(ols)}}$ shown in (27), and its *p*-th coordinate is

40 $$ {}{\begin{aligned} \beta_{ip}^{\text{(ols)}} &= \left[\left(\boldsymbol{\tilde{X}}^{\mathrm{T}} \boldsymbol{\tilde{X}}\right)^{-1} \boldsymbol{\tilde{X}}^{\mathrm{T}} \boldsymbol{\tilde{y}}_{i}\right]_{p} + \delta_{p} \\&= \frac{\boldsymbol{\tilde{y}}_{i}^{\mathrm{T}} \left[ \boldsymbol{I}_{n} - {\boldsymbol{\tilde{X}}^{-}} \left({\boldsymbol{\tilde{X}}^{-}}^{\mathrm{T}} {\boldsymbol{\tilde{X}}^{-}}\right)^{-1} {\boldsymbol{\tilde{X}}^{-}}^{\mathrm{T}} \right] \boldsymbol{\tilde{x}}^{p}}{{\boldsymbol{\tilde{x}}^{p}}^{\mathrm{T}} \left[ \boldsymbol{I}_{n} - {\boldsymbol{\tilde{X}}^{-}} \left({\boldsymbol{\tilde{X}}^{-}}^{\mathrm{T}} {\boldsymbol{\tilde{X}}^{-}}\right)^{-1} {\boldsymbol{\tilde{X}}^{-}}^{\mathrm{T}} \right] \boldsymbol{\tilde{x}}^{p}} + \delta_{p}, \end{aligned}}  $$

where the second equality follows from $\boldsymbol {\tilde {X}}= \begin {bmatrix} \boldsymbol {\tilde {X}}^{-} & \boldsymbol {x}^{p} \\ \end {bmatrix}$ and the inverse formula for the partitioned matrix of $\boldsymbol {\tilde {X}}^{\mathrm {T}} \boldsymbol {\tilde {X}}$. The value of *h*_*i*_(***β***_*i*_) at $\boldsymbol {\beta }_{i}=\boldsymbol {\beta }_{i}^{\text {(ols)}}$ is

41 $$ h_{i}\left(\boldsymbol{\beta}_{i}^{\text{(ols)}}\right) = \frac{1}{2\sigma_{i}^{2}} \boldsymbol{\tilde{y}}_{i}^{\mathrm{T}} \left[ \boldsymbol{I}_{n} - \boldsymbol{\tilde{X}} \left(\boldsymbol{\tilde{X}}^{\mathrm{T}} \boldsymbol{\tilde{X}}\right)^{-1} \boldsymbol{\tilde{X}}^{\mathrm{T}} \right] \boldsymbol{\tilde{y}}_{i} + \lambda_{i}.  $$

The decrease in the objective value from *β*_*ip*_=0 in Eq. (38) to *β*_*ip*_≠0 in Eq. (41) is

42 $$ {}{\begin{aligned} &h_{i}\left(\boldsymbol{\beta}_{i}^{\text{(r)}}\right) - h_{i}\left(\boldsymbol{\beta}_{i}^{\text{(ols)}}\right)\\ &=\; \frac{1}{2\sigma_{i}^{2}} \left\{ \boldsymbol{\tilde{y}}_{i}^{\mathrm{T}} \left[ \boldsymbol{\tilde{X}} \left(\boldsymbol{\tilde{X}}^{\mathrm{T}} \boldsymbol{\tilde{X}}\right)^{-1} \boldsymbol{\tilde{X}}^{\mathrm{T}} - {\boldsymbol{\tilde{X}}^{-}} \left({\boldsymbol{\tilde{X}}^{-}}^{\mathrm{T}} {\boldsymbol{\tilde{X}}^{-}}\right)^{-1} {\boldsymbol{\tilde{X}}^{-}}^{\mathrm{T}} \right] \boldsymbol{\tilde{y}}_{i} \; \right. \\ &+\;2 \boldsymbol{\tilde{y}}_{i}^{\mathrm{T}} \left[ \boldsymbol{I}_{n} - {\boldsymbol{\tilde{X}}^{-}} \left({\boldsymbol{\tilde{X}}^{-}}^{\mathrm{T}} {\boldsymbol{\tilde{X}}^{-}}\right)^{-1} {\boldsymbol{\tilde{X}}^{-}}^{\mathrm{T}} \right] \boldsymbol{\tilde{x}}^{p} \delta_{p} \;+ \\ \;&\left. {\boldsymbol{\tilde{x}}^{p}}^{\mathrm{T}} \left[ \boldsymbol{I}_{n} - {\boldsymbol{\tilde{X}}^{-}} \left({\boldsymbol{\tilde{X}}^{-}}^{\mathrm{T}} {\boldsymbol{\tilde{X}}^{-}}\right)^{-1} {\boldsymbol{\tilde{X}}^{-}}^{\mathrm{T}} \right] \boldsymbol{\tilde{x}}^{p} \delta_{p}^{2} \right\} - \lambda_{i} \\ =\;& \frac{1}{2\sigma_{i}^{2}} {\boldsymbol{\tilde{x}}^{p}}^{\mathrm{T}} \left[ \boldsymbol{I}_{n} - {\boldsymbol{\tilde{X}}^{-}} \left({\boldsymbol{\tilde{X}}^{-}}^{\mathrm{T}} {\boldsymbol{\tilde{X}}^{-}}\right)^{-1} {\boldsymbol{\tilde{X}}^{-}}^{\mathrm{T}} \right] \boldsymbol{\tilde{x}}^{p} \left|\beta_{ip}^{\text{(ols)}}\right|^{2} - \lambda_{i}, \end{aligned}}  $$

where the second equality employs the following equality:

$$\begin{aligned} &\boldsymbol{\tilde{y}}_{i}^{\mathrm{T}} \left[ \boldsymbol{\tilde{X}} \left(\boldsymbol{\tilde{X}}^{\mathrm{T}} \boldsymbol{\tilde{X}}\right)^{-1} \boldsymbol{\tilde{X}}^{\mathrm{T}} - {\boldsymbol{\tilde{X}}^{-}} \left({\boldsymbol{\tilde{X}}^{-}}^{\mathrm{T}} {\boldsymbol{\tilde{X}}^{-}}\right)^{-1} {\boldsymbol{\tilde{X}}^{-}}^{\mathrm{T}} \right] \boldsymbol{\tilde{y}}_{i} \\ = &\frac{\left\{\boldsymbol{\tilde{y}}_{i}^{\mathrm{T}} \left[ \boldsymbol{I}_{n} - {\boldsymbol{\tilde{X}}^{-}} \left({\boldsymbol{\tilde{X}}^{-}}^{\mathrm{T}} {\boldsymbol{\tilde{X}}^{-}}\right)^{-1} {\boldsymbol{\tilde{X}}^{-}}^{\mathrm{T}} \right] \boldsymbol{\tilde{x}}^{p}\right\}^{2}}{{\boldsymbol{\tilde{x}}^{p}}^{\mathrm{T}} \left[ \boldsymbol{I}_{n} - {\boldsymbol{\tilde{X}}^{-}} \left({\boldsymbol{\tilde{X}}^{-}}^{\mathrm{T}} {\boldsymbol{\tilde{X}}^{-}}\right)^{-1} {\boldsymbol{\tilde{X}}^{-}}^{\mathrm{T}} \right] \boldsymbol{\tilde{x}}^{p}}, \end{aligned} $$

which is obtained by partitioning $\boldsymbol {\tilde {X}}^{\mathrm {T}} \boldsymbol {\tilde {X}}$ into a 2×2 block matrix and then substituting the formula for its inverse, and $\beta _{ip}^{\text {(ols)}}$ is defined in Eq. (40).

Therefore, the solution is

43 $$ {}{\begin{aligned} \beta_{ip} = \left\{ \begin{array}{ll} 0 &\text{ if} \frac{1}{2\sigma_{i}^{2}} {\boldsymbol{\tilde{x}}^{p}}^{\mathrm{T}} \left[ \boldsymbol{I}_{n} - {\boldsymbol{\tilde{X}}^{-}} \left({\boldsymbol{\tilde{X}}^{-}}^{\mathrm{T}} {\boldsymbol{\tilde{X}}^{-}}\right)^{-1} {\boldsymbol{\tilde{X}}^{-}}^{\mathrm{T}} \right] \boldsymbol{\tilde{x}}^{p} \left|\beta_{ip}^{\text{(ols)}}\right|^{2} < \lambda_{i} \\ \beta_{ip}^{\text{(ols)}} &\text{ otherwise} \end{array}\right. \end{aligned}}  $$

Now we only need to solve for *δ*_*p*_. We have

44 $$ \begin{aligned} \hat{\delta}_{p} &= \arg \,\min_{\delta_{p}} \sum_{i=1}^{m} \min \left\{h_{i}\left(\boldsymbol{\beta}_{i}^{\text{(r)}}\right), h_{i}\left(\boldsymbol{\beta}_{i}^{\text{(ols)}}\right)\right\} \\ &= \arg \,\min_{\delta_{p}} \sum_{i=1}^{m} \min \left\{h_{i}\left(\boldsymbol{\beta}_{i}^{\text{(r)}}\right) - h_{i}\left(\boldsymbol{\beta}_{i}^{\text{(ols)}}\right), 0\right\}, \end{aligned}  $$

where the second equality is due to the fact that $h_{i}\left (\boldsymbol {\beta }_{i}^{\text {(ols)}}\right)$ is a constant independent of ***δ***.

Substituting (42) into (44) yields

45 $$ {}{\begin{aligned} \hat{\delta}_{p} &= \arg \,\min_{\delta_{p}} \sum_{i=1}^{m} \min\\ &\left\{\frac{1}{2\sigma_{i}^{2}} {\boldsymbol{\tilde{x}}^{p}}^{\mathrm{T}} \left[ \boldsymbol{I}_{n} - {\boldsymbol{\tilde{X}}^{-}} \left({\boldsymbol{\tilde{X}}^{-}}^{\mathrm{T}} {\boldsymbol{\tilde{X}}^{-}}\right)^{-1} {\boldsymbol{\tilde{X}}^{-}}^{\mathrm{T}} \right] \boldsymbol{\tilde{x}}^{p} \left|\beta_{ip}^{\text{(ols)}} \left(\delta_{p}\right)\right|^{2} - \lambda_{i}, 0 \right\}\\ &= \arg \,\min_{\delta_{p}} \sum_{i=1}^{m} \min\\& \left\{ \frac{1}{2\sigma_{i}^{2}} {\boldsymbol{\tilde{x}}^{p}}^{\mathrm{T}} \left[ \boldsymbol{I}_{n} - {\boldsymbol{\tilde{X}}^{-}} \left({\boldsymbol{\tilde{X}}^{-}}^{\mathrm{T}} {\boldsymbol{\tilde{X}}^{-}}\right)^{-1} {\boldsymbol{\tilde{X}}^{-}}^{\mathrm{T}} \right] \boldsymbol{\tilde{x}}^{p} \left|\beta_{ip}^{\text{(ols)}} \left(\delta_{p}\right)\right|^{2}, \lambda_{i} \right\}, \end{aligned}}  $$

where the $\beta _{ip}^{\text {(ols)}} \left (\delta _{p}\right)$ as a function of *δ*_*p*_ is defined in Eq. (40).

After $\hat {\delta }_{p}$ is estimated, the estimate of *β*_*ip*_ is obtained by substituting $\delta _{p}=\hat {\delta }_{p}$ into (43). Algorithm 2 describes the complete model fitting procedure.


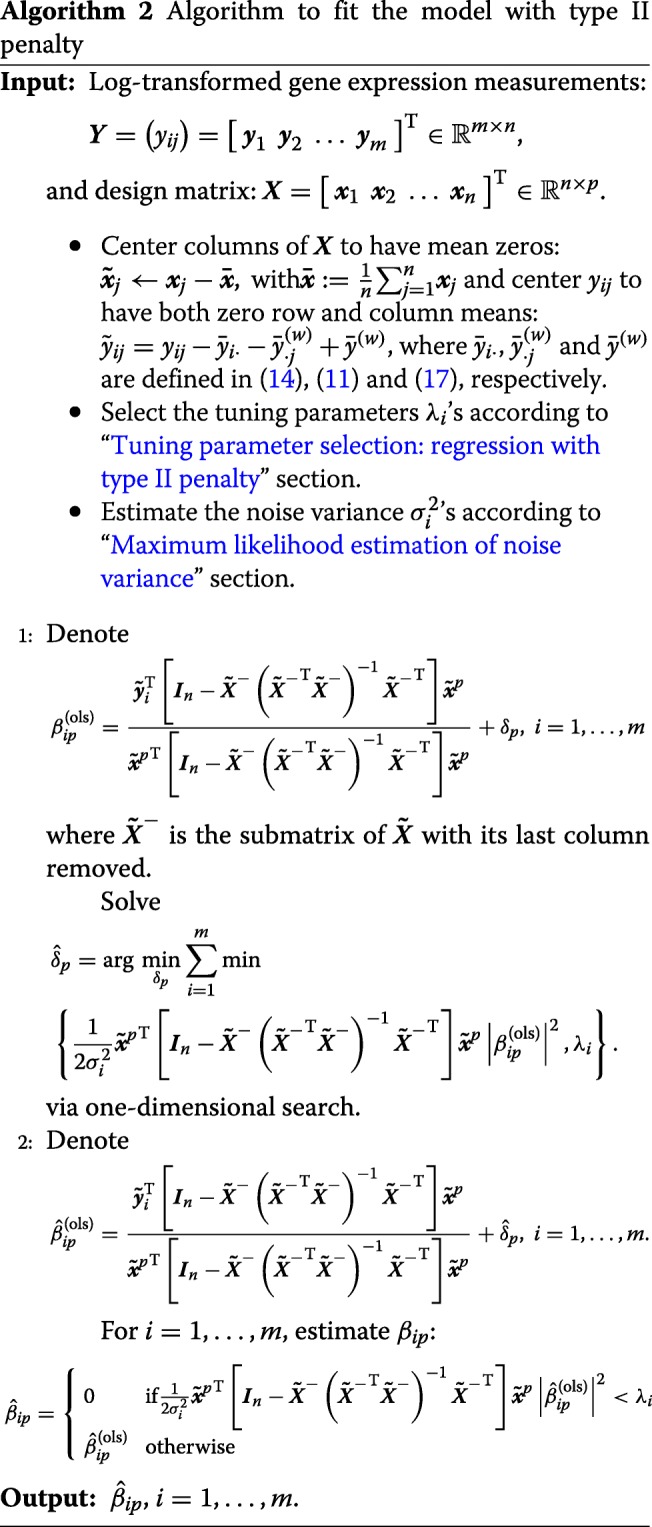


Next, we introduce a simple method for the selection of the tuning parameters in our model, which is based on the property of the solution (32) or (43).

#### Tuning parameter selection: regression with type I penalty

Substituting (19) into (1) and assuming that $\boldsymbol {\delta }=\boldsymbol {\bar {\beta }}^{(w)}$ is fixed, we have

46 $$ \tilde{y}_{ij} + \boldsymbol{\delta}^{\mathrm{T}} \boldsymbol{\tilde{x}}_{j} = \boldsymbol{\beta}_{i}^{\mathrm{T}} \boldsymbol{\tilde{x}}_{j} + \varepsilon_{ij},  $$

where $\tilde {y}_{ij} + \boldsymbol {\delta }^{\mathrm {T}} \boldsymbol {\tilde {x}}_{j} $ are the normalized data, which we use here as the response variables, and $\varepsilon _{ij} \sim \mathcal {N}\left (0,\sigma _{i}^{2}\right)$.

The condition for ***β***_*i*_=0 in (32) can be rewritten as

47 $$ \frac{\left\|\boldsymbol{\tilde{X}} \left[\left(\boldsymbol{\tilde{X}}^{\mathrm{T}} \boldsymbol{\tilde{X}}\right)^{-1} \boldsymbol{\tilde{X}}^{\mathrm{T}} \boldsymbol{\tilde{y}}_{i} + \boldsymbol{\delta}\right]\right\|^{2}}{\sigma_{i}^{2}} < 2\lambda_{i}.  $$

Under the null hypothesis, ***β***_*i*_=***0***; the left-hand side of (47) follows a chi-squared distribution with *p* degrees of freedom, i.e., $\chi _{p}^{2}$. This suggests us choose *λ*_*i*_=1/2·*F*^−1^(1−*q*; *p*)=1/2·{*x*:*F*(*x*; *p*)=1−*q*}, where *F*(*x*; *p*) is the cumulative distribution function of $\chi _{p}^{2}$, and *q* is a pre-specified significance level.

#### Tuning parameter selection: regression with type II penalty

Let $\tilde {y}_{ij} + \boldsymbol {\delta }^{\mathrm {T}} \boldsymbol {\tilde {x}}_{j} $ denote the normalized data:

48 $$ \tilde{y}_{ij} + \boldsymbol{\delta}^{\mathrm{T}} \boldsymbol{\tilde{x}}_{j} = \boldsymbol{\beta}_{i}^{\mathrm{T}} \boldsymbol{\tilde{x}}_{j} + \varepsilon_{ij},  $$

where $\varepsilon _{ij} \sim \mathcal {N}\left (0,\sigma _{i}^{2}\right)$.

The condition for *β*_*ip*_=0 in (43) can be rewritten as

49 $$ \left|\frac{\beta_{ip}^{\text{(ols)}}}{\text{SE}_{\beta_{ip}^{\text{(ols)}}}}\right| < \sqrt{2\lambda_{i}},  $$

where $\beta _{ip}^{\text {(ols)}}$ is defined in (40) and

$$ \text{SE}_{\beta_{ip}^{\text{(ols)}}} = \sqrt{ \frac{\sigma_{i}^{2}}{{\boldsymbol{\tilde{x}}^{p}}^{\mathrm{T}} \left[ \boldsymbol{I}_{n} - {\boldsymbol{\tilde{X}}^{-}} \left({\boldsymbol{\tilde{X}}^{-}}^{\mathrm{T}} {\boldsymbol{\tilde{X}}^{-}}\right)^{-1} {\boldsymbol{\tilde{X}}^{-}}^{\mathrm{T}} \right] \boldsymbol{\tilde{x}}^{p}} } $$

is the standard error of the estimate $\beta _{ip}^{\text {(ols)}}$.

Under the null hypothesis, *β*_*ip*_=0; the left-hand side of (49) follows the standard Gaussian distribution. This suggests us choose *λ*_*i*_=1/2·[*Φ*^−1^(1−*q*/2)]^2^, where *Φ*(·) is the cumulative distribution function of the standard Gaussian distribution, and *q* is a pre-specified significance level.

#### Maximum likelihood estimation of noise variance

To estimate $\sigma _{i}^{2}, i=1,\dots,m$, consider the negative log-likelihood function with $\sigma _{i}^{2}$’s being unknown as well:

50 $$ {}{\begin{aligned} &l\left(\boldsymbol{\alpha}, \{\boldsymbol{\beta}_{i}\}_{i=1}^{m}, \{\sigma_{i}^{2}\}_{i=1}^{m}, \boldsymbol{d}\right) \\&= {\sum_{i=1}^{m}} \left[ \frac{n}{2} \log(2\pi\sigma_{i}^{2}) + \frac{1}{2\sigma_{i}^{2}} {\sum_{j=1}^{n}} \left(y_{ij}-\alpha_{i}-\boldsymbol{x}_{j}^{\mathrm{T}} \boldsymbol{\beta}_{i}-d_{j}\right)^{2} \right]. \end{aligned}}  $$

Setting partial derivatives of *l*(·) with respect to *α*_*i*_,***β***_*i*_,*i*=1,…,*m*, and *d*_*j*_,*j*=1,…,*n* to zeros, and after some mathematical manipulation, we obtain

51 $$ \boldsymbol{\beta}_{i} = \left(\boldsymbol{\tilde{X}}^{\mathrm{T}} \boldsymbol{\tilde{X}}\right)^{-1} \sum_{j=1}^{n} \boldsymbol{\tilde{x}}_{j} \left(y_{ij} - \bar{y}_{\cdot j}^{(w)}\right) + \boldsymbol{\bar{\beta}}^{(w)},  $$

where $\boldsymbol {\tilde {X}}, \boldsymbol {\tilde {x}}_{j}$ and $\bar {y}_{\cdot j}^{(w)}$ are defined in (28), (22) and (11), respectively.

Taking partial derivatives of *l*(·) with respect to $\sigma _{i}^{2}, i=1,\ldots,m,$ and setting them to zeros gives

52 $$ \sigma_{i}^{2} = \frac{1}{n} {\sum_{j=1}^{n}} \left(y_{ij}-\alpha_{i}-\boldsymbol{x}_{j}^{\mathrm{T}} \boldsymbol{\beta}_{i}-d_{j}\right)^{2}.  $$

Substituting (19) into (52), we have

53 $$ \sigma_{i}^{2} = \frac{1}{n} {\sum_{j=1}^{n}} \left(y_{ij} - \bar{y}_{i\cdot} - \bar{y}_{\cdot j}^{(w)} + \bar{y}^{(w)} - \boldsymbol{\tilde{x}}_{j}^{\mathrm{T}} \boldsymbol{\beta}_{i} + \boldsymbol{\tilde{x}}_{j}^{\mathrm{T}} \boldsymbol{\bar{\beta}}^{(w)} \right)^{2},  $$

where $\bar {y}_{i\cdot }$ and $\bar {y}^{(w)}$ are as defined in (14) and (17), respectively.

Given initial estimates of $\sigma _{i}^{2}$’s and $\boldsymbol {\bar {\beta }}^{(w)}, \{\boldsymbol {\beta }_{i}\}, \{\sigma _{i}^{2}\}$ and $\boldsymbol {\bar {\beta }}^{(w)}$ can be updated alternately using Eqs. (51), (53), and (12) until convergence.

After $\{\sigma _{i}^{2}\}_{i=1}^{m}$ are estimated, they can be robustified using a “shrinkage toward the mean” scheme [16]:

54 $$ \hat{\hat{\sigma}}_{i}^{2}=(1-w) \hat{\sigma}_{i}^{2}+w\overline{\hat{\sigma}^{2}}  $$

where

55 $$ \overline{\hat{\sigma}^{2}}= \frac{1}{m} \sum_{i=1}^{m} \hat{\sigma}_{i}^{2},  $$

56 $$ w=\frac{2(m-1)}{n-p+1}\left(\frac{1}{m}+\frac{(\overline{\hat{\sigma}^{2}})^{2}}{\sum_{i=1}^{m}\left(\hat{\sigma}_{i}^{2}-\overline{\hat{\sigma}^{2}}\right)^{2}}\right).  $$

The noise variance estimates $\hat {\hat {\sigma }}_{i}^{2}, i=1,\dots,m$, can then be used in Algorithm 1 or 2 to solve for $\{\boldsymbol {\beta }_{i}\}_{i=1}^{m}$.

##### **Remark 2**

Note that when $ \sigma _{1}^{2} = \sigma _{2}^{2} = \dotsb = \sigma _{m}^{2} = \sigma ^{2} $, it is no longer needed to estimate *σ*^2^ since *σ*^2^ in (6) can be incorporated into the tuning parameters *λ*_*i*_’s.

## Results and discussion

We demonstrate the performance of our proposed method (named rSeqRobust) by comparing it with other existing methods for DE gene detection from RNA-seq data: edgeR-robust [1*,*^2^*], DESeq2 [*3*], limma-voom [*4*,*^5^], and ELMSeq (which fits a similar model but with *ℓ*_1_ rather than *ℓ*_0_ penalty) [11]. We consider the simple regression model (*p*=1), in which case Algorithms 1 and 2 coincide. For ELMSeq, the tuning parameter is set as the 5th percentile of *m* values: $\left |\frac {1}{\hat {\sigma }_{1}^{2}} \boldsymbol {\tilde {x}}^{\mathrm {T}} \boldsymbol {\tilde {y}}_{1}\right |, \left |\frac {1}{\hat {\sigma }_{2}^{2}} \boldsymbol {\tilde {x}}^{\mathrm {T}} \boldsymbol {\tilde {y}}_{2}\right |, \dots, \left |\frac {1}{\hat {\sigma }_{m}^{2}} \boldsymbol {\tilde {x}}^{\mathrm {T}} \boldsymbol {\tilde {y}}_{m}\right |$ [11]. The tuning parameters *λ* is set based on the significant level of *q*=0.01.

### Simulations on synthetic data

We simulate both log-normally distributed and negative-binomially distributed read counts, with *m*=20,000 genes and *n*=7, 20 or 200 samples. The RNA-seq read counts are generated as $c_{ij} \sim \lceil \mathrm {e}^{\mathcal {N}(\log \mu _{ij},\sigma _{i}^{2})}\rceil $ under the log-normal (LN) distribution assumption, and as $c_{ij} \sim \mathcal {NB}(\mu _{ij},\phi _{i})$ [2] under the NB distribution assumption, where *μ*_*ij*_ is the mean read counts of gene *i* in sample *j*. The generation of *μ*_*ij*_ is described in Table 1. The variance of the normal distribution is set as $\sigma _{i}^{2}=0.01$, and the dispersion parameter of the NB distribution is set as *ϕ*_*i*_=0.25.

**Table 1:** Models and parameters for synthetic data generation

*ℓ*_*i*_∼e^unif(5,10)^	length of gene *i*
$\alpha _{i} \sim \mathcal {N}(0,1)$	other log scaling factors of gene *i*
*β*_*i*_=0	log-fold change for non-DE genes
$\beta _{i} \sim \mathcal {N}(2,1)$	log-fold change for up-regulated DE genes
$\beta _{i} \sim \mathcal {N}(-2,1)$	log-fold change for down-regulated DE genes
$x_{j} \sim \mathcal {N}(0,1)$	covariates for sample *j*
*N*_*j*_∼unif(2,3)×10^6^	library size of sample *j*
$d_{j} \sim \mathcal {N}(0, 1)$	other log scaling factors of sample *j*
$\mu _{ij} = N_{j} \frac {\ell _{i}}{\sum _{i=1}^{m} \ell _{i}} \mathrm {e}^{\alpha _{i} + \beta _{i} x_{j} + d_{j}}$	mean read counts of gene *i* in sample *j*

After estimating the sample-specific normalization factors *d*_*j*_’s using Algorithm 1, we substitute $\hat {d}_{j}$’s into model (1) to obtain *m* decoupled gene-wise linear regression models. For each gene *i*, we test the null hypothesis that *β*_*i*_=0, and calculate the *p*-value. We decide there is a significant linear association between the experimental variable *x*_*j*_ and the gene expression *y*_*ij*_ if the *p*-value is less than a predefined threshold (e.g., 0.05). With the *p*-value for each gene, we rank the genes and vary the *p*-value threshold from 0 to 1 to determine significant DE genes and calculate the area under the ROC curve (AUC).

Table 2 shows the AUCs of the five methods on log-normally distributed data with sample size *n*=20. We observe the followings: i) In relatively easy scenarios where a small percent of genes are differentially expressed (i.e., DE% =1% or 10%) or the up- and down-regulated genes are equal in portions (i.e., Up% =50%), all five methods perform equally well (within one standard error of AUC difference); ii) In challenging scenarios where a large percent of genes are differentially expressed (i.e., DE% ≥30%) and the up- and down-regulated genes are different in portions (i.e., Up% =75% or 100%), rSeqRobust outperforms ELMSeq, which in turn outperforms the rest; iii) In the most challenging scenarios with 70% or 90% DE genes that are all overexpressed (i.e., Up% =100%), only rSeqRobust achieves good results (AUC=0.9638 or 0.8276).

**Table 2:** The AUCs of edgeR-robust, DESeq2, limma-voom, ELMSeq and rSeqRobust based on log-normally distributed data

DE (%)	Up (%)	edgeR	DESeq2	voom	ELMSeq	rSeqRobust
1	50	0.9734	0.9736	**0.9786**	0.9717	0.9757
		(0.0091)	(0.009)	**(0.007)**	(0.0065)	(0.0064)
1	75	0.954	0.9531	**0.9711**	0.9343	0.935
		(0.0113)	(0.0141)	**(0.0086)**	(0.0153)	(0.018)
1	100	0.9525	0.9531	**0.9633**	0.9476	0.9594
		(0.0144)	(0.0137)	**(0.0108)**	(0.0151)	(0.0139)
10	50	0.958	0.9623	**0.9668**	0.9573	0.9627
		(0.0079)	(0.0069)	**(0.0057)**	(0.0069)	(0.0067)
10	75	0.9707	0.9632	**0.9749**	0.964	0.9668
		(0.0057)	(0.0045)	**(0.004)**	(0.0061)	(0.0057)
10	100	0.9403	0.9272	**0.9605**	0.94	0.9435
		(0.0107)	(0.0142)	**(0.0077)**	(0.0128)	(0.0128)
30	50	0.9689	0.9696	**0.974**	0.9665	0.9678
		(0.0056)	(0.0048)	**(0.005)**	(0.0053)	(0.0052)
30	75	0.9318	0.9265	0.9458	0.9564	**0.9655**
		(0.0113)	(0.0116)	(0.0096)	(0.0078)	**(0.0059)**
30	100	0.8771	0.8693	0.8753	0.9372	**0.9566**
		(0.0153)	(0.0091)	(0.0145)	(0.0149)	**(0.0086)**
50	50	0.9466	0.954	0.9425	0.9557	**0.957**
		(0.0092)	(0.0059)	(0.0087)	(0.0071)	**(0.0065)**
50	75	0.9099	0.906	0.9145	0.9401	**0.9566**
		(0.0167)	(0.0123)	(0.0178)	(0.0135)	**(0.0076)**
50	100	0.7083	0.7236	0.7197	0.879	**0.9576**
		(0.022)	(0.0291)	(0.0242)	(0.0195)	**(0.0071)**
70	50	0.967	0.9655	0.9652	0.9655	**0.969**
		(0.0039)	(0.0034)	(0.0036)	(0.0031)	**(0.0021)**
70	75	0.8569	0.8351	0.8564	0.9089	**0.9692**
		(0.0193)	(0.0161)	(0.0194)	(0.0118)	**(0.0045)**
70	100	0.4536	0.5212	0.4893	0.4786	**0.9638**
		(0.0344)	(0.0296)	(0.018)	(0.037)	**(0.0082)**
90	50	0.953	0.9538	0.9513	**0.9561**	0.9512
		(0.0064)	(0.0064)	(0.0081)	**(0.0042)**	(0.0049)
90	75	0.7203	0.6918	0.7256	0.6906	**0.9584**
		(0.0239)	(0.0177)	(0.0323)	(0.0167)	**(0.0084)**
90	100	0.2568	0.506	0.2566	0.3516	**0.8276**
		(0.0257)	(0.0265)	(0.0278)	(0.0345)	**(0.0426)**

The sample size is *n*=20. The variance of the normal distribution is $\sigma _{i}^{2}=0.01$. The table shows the percent of DE genes (DE %), percent of up-regulated genes among all the DE genes (Up %), and the mean AUCs (standard errors in parentheses) for all five methods with 10 simulated replicates. The highest AUC value is shown in bold

Table 3 shows the AUCs of different methods for negative-binomially distributed data. The same observations are obtained as in Table 2: In relatively easy settings with small percent of DE genes or symmetric over- and under-expression pattern, rSeqRobust performs as well as other methods; In challenging settings with large percent of DE genes (DE% ≥10%) and asymmetric over- and under-expression pattern (Up% =75% or 100%), rSeqRobust consistently performs the best, and ELMSeq ranks second in all except extreme cases: (Up%, DE%) =(70%, 100%), (90%, 75%) or (90%, 100%) where most methods suffer from severe performance degradation or complete failure.

**Table 3:** The AUCs of edgeR-robust, DESeq2, limma-voom, ELMSeq and rSeqRobust based on negative-binomially distributed data

DE (%)	Up (%)	edgeR	DESeq2	voom	ELMSeq	rSeqRobust
1	50	0.9585	0.9635	**0.9636**	0.9636	0.9622
		(0.0105)	(0.0105)	**(0.0101)**	(0.0106)	(0.0112)
1	75	0.9644	0.9696	0.967	0.9711	**0.9734**
		(0.0114)	(0.0098)	(0.0105)	(0.0095)	**(0.0088)**
1	100	**0.9785**	0.9711	0.977	0.9765	0.9754
		**(0.0051)**	(0.0083)	(0.0061)	(0.005)	(0.0056)
10	50	0.9576	0.9604	0.9613	0.9647	**0.9658**
		(0.005)	(0.0035)	(0.0039)	(0.0042)	**(0.0036)**
10	75	0.9551	0.957	0.9559	0.9613	**0.9664**
		(0.0054)	(0.0075)	(0.0061)	(0.0075)	**(0.0047)**
10	100	0.9469	0.9496	0.9474	0.9611	**0.9635**
		(0.0105)	(0.008)	(0.0103)	(0.0059)	**(0.0056)**
30	50	0.9509	0.9528	0.949	**0.9604**	0.9582
		(0.0083)	(0.0045)	(0.0101)	**(0.0035)**	(0.0043)
30	75	0.9413	0.9428	0.9406	0.9664	**0.9673**
		(0.0093)	(0.0056)	(0.0069)	(0.0026)	**(0.0024)**
30	100	0.8689	0.8629	0.879	0.9128	**0.9429**
		(0.015)	(0.0106)	(0.0168)	(0.0113)	**(0.0061)**
50	50	0.9599	0.9618	0.9543	**0.9629**	0.962
		(0.0081)	(0.006)	(0.0086)	**(0.0054)**	(0.006)
50	75	0.8834	0.8902	0.892	0.9279	**0.9482**
		(0.0123)	(0.0131)	(0.01)	(0.0132)	**(0.0078)**
50	100	0.7465	0.7003	0.7425	0.8802	**0.9595**
		(0.0302)	(0.0174)	(0.0318)	(0.012)	**(0.0058)**
70	50	0.9565	0.9629	0.956	**0.9637**	0.9636
		(0.0049)	(0.0036)	(0.0054)	**(0.0025)**	(0.0026)
70	75	0.8164	0.7922	0.8264	0.8847	**0.956**
		(0.0187)	(0.0066)	(0.0248)	(0.0107)	**(0.0033)**
70	100	0.4964	0.488	0.5462	0.4482	**0.9522**
		(0.0323)	(0.0227)	(0.0315)	(0.0224)	**(0.0046)**
90	50	0.9503	**0.9604**	0.9463	0.9584	0.9478
		(0.0064)	**(0.0037)**	(0.0077)	(0.0037)	(0.0062)
90	75	0.6657	0.6272	0.6879	0.5992	**0.6912**
		(0.0205)	(0.0124)	(0.0226)	(0.0131)	**(0.0946)**
90	100	0.2455	0.4752	0.2905	0.2826	**0.5379**
		(0.0317)	(0.0225)	(0.0316)	(0.0214)	**(0.1178)**

The table shows the percent of DE genes (DE %), percent of up-regulated genes among all the DE genes (Up %), and the mean AUCs (standard errors in parentheses) for all five methods with 10 simulated replicates. The highest AUC value is shown in bold

In Tables 4 and 5, the sample size is reduced to *n*=7. Again, we observe similar patterns: when a small subset of genes are differentially expressed (i.e., DE% =1% or 10%), or the up- and down-regulated DE genes are imbalanced in numbers, rSeqRobust and other methods perform equally well; when most genes are differentially expressed (i.e., DE% = 50% or 70%) in an asymmetric manner (i.e., Up% =75% or 100%), rSeqRobust outperforms all other methods. Note that in the presence of 70% DE genes that are all up-regulated, only rSeqRobust achieves good results (AUC =0.9059 for LN data and AUC =0.8623 for NB data).

**Table 4:** The AUCs of edgeR-robust, DESeq2, limma-voom, ELMSeq and rSeqRobust based on log-normally distributed data

DE (%)	Up (%)	edgeR - robust	DESeq2	limma - voom	ELMSeq	rSeqRobust
1	50	0.9349	**0.9442**	0.9087	0.9243	0.9277
		(0.0222)	**(0.0134)**	(0.0265)	(0.0154)	(0.0156)
1	75	0.9349	0.9423	**0.9436**	0.9359	0.9315
		(0.0153)	(0.0125)	**(0.0144)**	(0.015)	(0.0147)
1	100	0.907	0.8781	**0.9235**	0.8498	0.8481
		(0.0391)	(0.0456)	**(0.0398)**	(0.0579)	(0.0596)
10	50	0.8743	**0.8772**	0.8687	0.8604	0.864
		(0.0177)	**(0.0171)**	(0.0211)	(0.0194)	(0.0192)
10	75	0.9043	0.8916	**0.9275**	0.8751	0.8729
		(0.0256)	(0.0276)	**(0.0226)**	(0.0329)	(0.0373)
10	100	0.9217	0.8959	0.9174	0.9194	**0.9217**
		(0.0185)	(0.0191)	(0.0233)	(0.0201)	**(0.0205)**
30	50	0.9154	0.9111	**0.9196**	0.8874	0.8937
		(0.0141)	(0.0177)	**(0.0153)**	(0.023)	(0.0224)
30	75	**0.9021**	0.8762	0.8942	0.8777	0.8862
		**(0.0324)**	(0.0395)	(0.0407)	(0.0458)	(0.0509)
30	100	0.8599	0.8431	0.8658	0.8391	**0.8964**
		(0.0201)	(0.0175)	(0.022)	(0.0265)	**(0.0149)**
50	50	0.9018	**0.9178**	0.9035	0.8978	0.8914
		(0.0187)	**(0.0132)**	(0.0162)	(0.0162)	(0.0252)
50	75	0.8704	0.8681	0.8724	0.8719	**0.9066**
		(0.02)	(0.021)	(0.0182)	(0.027)	**(0.0215)**
50	100	0.7227	0.759	0.7251	0.8133	**0.8809**
		(0.0331)	(0.0278)	(0.0291)	(0.036)	**(0.0268)**
70	50	0.8804	**0.905**	0.8641	0.9004	0.8885
		(0.0247)	**(0.0238)**	(0.0348)	(0.0258)	(0.0301)
70	75	0.8073	0.8202	0.8088	**0.8761**	0.8747
		(0.0275)	(0.0285)	(0.0241)	**(0.0277)**	(0.0227)
70	100	0.4748	0.5097	0.4891	0.4778	**0.9059**
		(0.0507)	(0.0415)	(0.0601)	(0.0614)	**(0.0165)**
90	50	0.8905	**0.9316**	0.8625	0.9094	0.8581
		(0.0299)	**(0.0113)**	(0.0322)	(0.0116)	(0.0433)
90	75	0.6897	0.6534	0.7015	0.6706	**0.7144**
		(0.0485)	(0.0438)	(0.045)	(0.0379)	**(0.0721)**
90	100	0.2229	**0.4989**	0.2818	0.3102	0.411
		(0.04)	**(0.0297)**	(0.0365)	(0.041)	(0.0916)

The table shows the percent of DE genes (DE %), percent of up-regulated genes among all the DE genes (Up %), and the mean AUCs (standard errors in parentheses) for all five methods with 10 simulated replicates. The highest AUC value is shown in bold

**Table 5:** The AUCs of edgeR-robust, DESeq2, limma-voom, ELMSeq and rSeqRobust based on negative-binomially distributed data

DE (%)	Up (%)	edgeR - robust	DESeq2	limma - voom	ELMSeq	rSeqRobust
1	50	0.8696	0.8944	0.8686	0.8924	**0.9052**
		(0.0378)	(0.0175)	(0.0389)	(0.017)	**(0.0162)**
1	75	**0.9085**	0.9038	0.8961	0.9001	0.9057
		**(0.0166)**	(0.0146)	(0.0166)	(0.0163)	(0.0162)
1	100	0.9108	0.898	**0.9158**	0.8992	0.8933
		(0.0228)	(0.0279)	**(0.0205)**	(0.0223)	(0.0237)
10	50	**0.9189**	0.9176	0.9141	0.9092	0.9126
		**(0.0089)**	(0.009)	(0.0091)	(0.0091)	(0.008)
10	75	**0.9025**	0.8999	0.8994	0.8892	0.8961
		**(0.011)**	(0.0099)	(0.0124)	(0.0122)	(0.0108)
10	100	0.8558	**0.8656**	0.8646	0.854	0.8651
		(0.0263)	**(0.0257)**	(0.0217)	(0.029)	(0.0258)
30	50	**0.9156**	0.9148	0.9082	0.9046	0.9037
		**(0.0117)**	(0.0108)	(0.0126)	(0.0097)	(0.0095)
30	75	0.8963	0.9002	0.8879	0.8935	**0.904**
		(0.0134)	(0.008)	(0.0171)	(0.0126)	**(0.0096)**
30	100	0.8655	0.8843	0.8489	0.8962	**0.9215**
		(0.02)	(0.0091)	(0.0244)	(0.0085)	**(0.0073)**
50	50	0.8924	**0.9006**	0.8804	0.8888	0.8895
		(0.0146)	**(0.012)**	(0.0201)	(0.0129)	(0.0123)
50	75	0.8837	0.9025	0.8761	0.8925	**0.9241**
		(0.0214)	(0.0095)	(0.0219)	(0.024)	**(0.0083)**
50	100	0.6974	0.6906	0.6963	0.7648	**0.854**
		(0.0255)	(0.0261)	(0.0236)	(0.029)	**(0.021)**
70	50	0.8985	**0.9097**	0.8948	0.8897	0.8806
		(0.0175)	**(0.0101)**	(0.0168)	(0.0144)	(0.0189)
70	75	0.7951	0.7845	0.806	0.8163	**0.8678**
		(0.0203)	(0.0094)	(0.0236)	(0.0158)	**(0.0253)**
70	100	0.5673	0.4875	0.5651	0.48	**0.8623**
		(0.0271)	(0.0255)	(0.0326)	(0.0261)	**(0.024)**
90	50	0.8809	**0.9014**	0.8658	0.8841	0.8025
		(0.0184)	**(0.0143)**	(0.0233)	(0.0169)	(0.0367)
90	75	0.6859	0.6557	**0.6886**	0.651	0.6562
		(0.0422)	(0.032)	**(0.0399)**	(0.0378)	(0.0565)
90	100	0.2348	0.3932	0.2105	0.2978	**0.4837**
		(0.0256)	(0.0273)	(0.0355)	(0.0196)	**(0.0576)**

The table shows the percent of DE genes (DE %), percent of up-regulated genes among all the DE genes (Up %), and the mean AUCs (standard errors in parentheses) for all five methods with 10 simulated replicates. The highest AUC value is shown in bold

In Tables 6 and 7, the sample size is increased to *n*=200. As the sample size increases from *n*=20 to *n*=200, the AUCs of edgeR-robust, DESeq2 and limma-voom increase for easy cases (small percent of DE genes or symmetric over- and under-expression patterns). However, for challenging cases (i.e., DE% =50%, 70% or 90%, Up% =75% or 100%), the AUCs decrease. On the contrary, the AUC of rSeqRobust increases consistently in all cases. The performance gain of rSeqRobust over other methods is more significant for more challenging cases. Note that rSeqRobust performs nearly as well in the most challenging cases (Up%, DE%) =(50%, 100%), (70%, 100%), (90%, 75%) or (90%, 100%) as in easy cases. In contrast, ELMSeq only works for (Up%, DE%) =(50%, 100%) and edgeR-robust, DESeq2, limma-voom completely fail in all these cases. This indicates that rSeqRobust is more robust than ELMSeq, which in turn is more robust than edgeR-robust, DESeq2 and limma-voom.

**Table 6:** The AUCs of edgeR-robust, DESeq2, limma-voom, ELMSeq and rSeqRobust based on log-normally distributed data

DE (%)	Up (%)	edgeR - robust	DESeq2	limma - voom	ELMSeq	rSeqRobust
1	50	0.9727	0.9861	**0.9906**	0.9864	0.9863
		(0.0077)	(0.0066)	**(0.0051)**	(0.0061)	(0.0063)
1	75	0.9951	**0.9994**	0.9991	0.9986	0.9991
		(0.0032)	**(4e-04)**	(9e-04)	(9e-04)	(8e-04)
1	100	0.9774	0.9892	**0.9939**	0.9811	0.9845
		(0.0089)	(0.0068)	**(0.0026)**	(0.0093)	(0.0135)
10	50	0.9807	0.9889	**0.989**	0.983	0.9847
		(0.0038)	(0.0016)	**(0.0021)**	(0.0026)	(0.0025)
10	75	0.9803	0.9856	0.9889	0.987	**0.9895**
		(0.0037)	(0.0027)	(0.0019)	(0.0023)	**(0.0028)**
10	100	0.9601	0.9568	**0.979**	0.9784	0.9763
		(0.0072)	(0.007)	**(0.0038)**	(0.0052)	(0.0073)
30	50	0.9811	**0.9886**	0.9878	0.9854	0.9864
		(0.002)	**(8e-04)**	(0.002)	(9e-04)	(0.001)
30	75	0.9321	0.946	0.9576	0.9836	**0.9856**
		(0.005)	(0.0036)	(0.0031)	(0.0026)	**(0.0026)**
30	100	0.8313	0.7859	0.8892	0.9725	**0.9809**
		(0.0217)	(0.0072)	(0.0171)	(0.0036)	**(0.0028)**
50	50	0.9836	**0.9904**	0.9856	0.9889	0.9893
		(0.002)	**(0.0016)**	(0.0013)	(0.0013)	(0.0013)
50	75	0.8518	0.8061	0.8857	0.9787	**0.987**
		(0.0218)	(0.011)	(0.0167)	(0.0024)	**(0.002)**
50	100	0.5708	0.5533	0.5863	0.896	**0.9827**
		(0.0356)	(0.0086)	(0.0223)	(0.0078)	**(0.0029)**
70	50	0.9763	**0.9875**	0.97	0.986	0.9871
		(0.0034)	**(0.0013)**	(0.0085)	(0.0022)	(0.0019)
70	75	0.7051	0.5986	0.7466	0.885	**0.9826**
		(0.0226)	(0.0139)	(0.0311)	(0.0109)	**(0.003)**
70	100	0.3702	0.5275	0.3727	0.3825	**0.9864**
		(0.0052)	(0.0097)	(0.013)	(0.0018)	**(0.0028)**
90	50	0.9792	0.9851	0.9766	0.9878	**0.9894**
		(0.0034)	(0.0027)	(0.0035)	(0.0019)	**(0.0016)**
90	75	0.4242	0.5324	0.4887	0.4061	**0.9869**
		(0.0163)	(0.0135)	(0.0205)	(0.0049)	**(0.0018)**
90	100	0.3881	0.5456	0.3553	0.3833	**0.9841**
		(0.003)	(0.0119)	(0.0027)	(0.0026)	**(0.0018)**

The table shows the percent of DE genes (DE %), percent of up-regulated genes among all the DE genes (Up %), and the mean AUCs (standard errors in parentheses) for all five methods with 10 simulated replicates. The highest AUC value is shown in bold

**Table 7:** The AUCs of edgeR-robust, DESeq2, limma-voom, ELMSeq and rSeqRobust based on negative-binomially distributed data

DE (%)	Up (%)	edgeR - robust	DESeq2	limma - voom	ELMSeq	rSeqRobust
1	50	0.9934	0.9919	0.9922	**0.9942**	0.9937
		(0.0038)	(0.0043)	(0.0048)	**(0.0039)**	(0.0045)
1	75	0.9933	0.9933	0.993	**0.9953**	0.9922
		(0.0033)	(0.0043)	(0.0036)	**(0.0032)**	(0.0047)
1	100	0.9882	0.9836	0.9867	**0.9901**	0.9891
		(0.0046)	(0.0057)	(0.0054)	**(0.0045)**	(0.0047)
10	50	0.9866	0.9892	0.9876	**0.9898**	0.9895
		(0.0024)	(0.0021)	(0.0024)	**(0.002)**	(0.0019)
10	75	0.9775	0.9803	0.9795	0.9867	**0.9874**
		(0.0037)	(0.0044)	(0.0032)	(0.0024)	**(0.0025)**
10	100	0.9724	0.9739	0.9788	0.9864	**0.9883**
		(0.0045)	(0.0046)	(0.0035)	(0.0032)	**(0.0028)**
30	50	0.9838	**0.9881**	0.9851	0.9874	0.9878
		(0.0022)	**(0.0018)**	(0.0022)	(0.0017)	(0.0014)
30	75	0.9568	0.9601	0.9614	0.9837	**0.9868**
		(0.0058)	(0.0022)	(0.0052)	(0.0023)	**(0.0015)**
30	100	0.8809	0.8902	0.89	0.9837	**0.9898**
		(0.0171)	(0.0044)	(0.0143)	(0.0014)	**(0.0013)**
50	50	0.982	**0.9875**	0.9823	0.9867	0.9869
		(0.0022)	**(0.0013)**	(0.0027)	(0.0017)	(0.0016)
50	75	0.9178	0.8977	0.9228	0.9799	**0.986**
		(0.008)	(0.0074)	(0.0069)	(0.0015)	**(0.0013)**
50	100	0.5817	0.5509	0.6413	0.9157	**0.9923**
		(0.0345)	(0.0104)	(0.027)	(0.0026)	**(0.0012)**
70	50	0.9811	**0.9873**	0.9807	0.9873	0.9871
		(0.0026)	**(0.0016)**	(0.0022)	(0.0013)	(0.0014)
70	75	0.7935	0.6559	0.8258	0.9108	**0.986**
		(0.0348)	(0.023)	(0.0306)	(0.0061)	**(0.0013)**
70	100	0.3529	0.4508	0.3866	0.3371	**0.9865**
		(0.0082)	(0.0222)	(0.0188)	(0.003)	**(0.0018)**
90	50	0.9842	0.9867	0.9849	0.987	**0.9875**
		(0.0023)	(0.0019)	(0.0019)	(0.0015)	**(0.0015)**
90	75	0.5017	0.5326	0.5683	0.4044	**0.9864**
		(0.0238)	(0.0121)	(0.0247)	(0.0104)	**(7e-04)**
90	100	0.3403	0.5145	0.2979	0.3167	**0.9828**
		(0.0033)	(0.0092)	(0.0021)	(0.003)	**(0.0012)**

The table shows the percent of DE genes (DE %), percent of up-regulated genes among all the DE genes (Up %), and the mean AUCs (standard errors in parentheses) for all five methods with 10 simulated replicates. The highest AUC value is shown in bold

Table 8 shows the average running times (in seconds) of the five methods on an Intel Core i3 processor with 8GB of memory and a clock frequency of 3.9GHz. We can see that rSeqRobust is slower than limma-voom; however, it scales well for large sample sizes and is much faster than ELMSeq.

**Table 8:** The computational times (in seconds) of edgeR-robust, DESeq2, limma-voom, ELMSeq and rSeqRobust

*n*	edgeR	DESeq2	voom	ELMSeq	rSeqRobust
7	5.45	0.76	**0.13**	403.39	16.63
20	9.49	1.51	**0.16**	987.87	21.84
200	70.68	49.30	**0.54**	2225.95	76.93

Percent of DE genes: 10%, percent of up-regulated genes among the DE genes: 50%. The least time is shown in bold

### Application to a real RNA-seq dataset

**Fig. 1:**
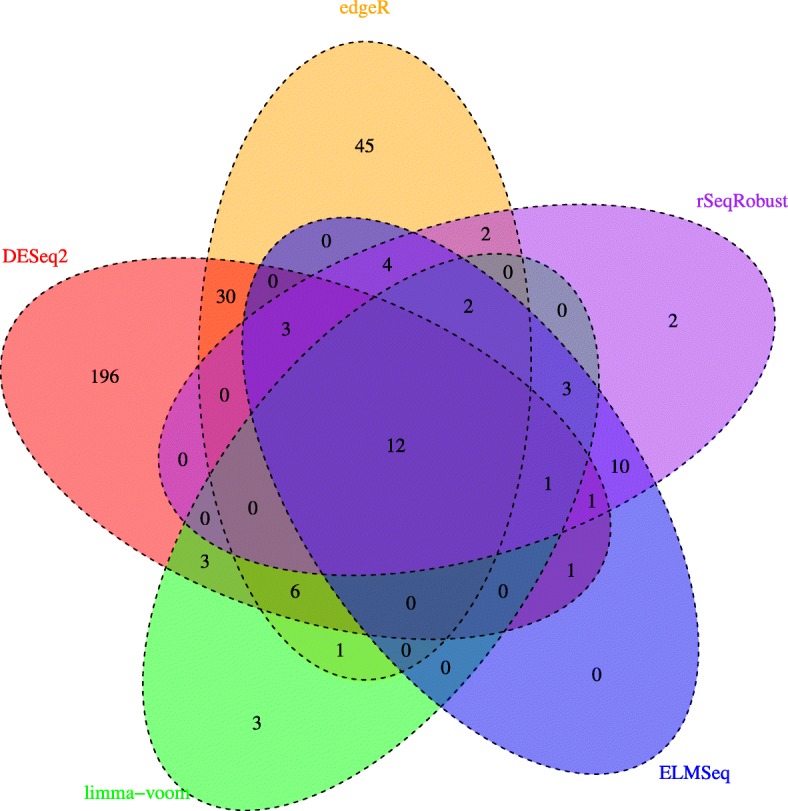
Venn diagram based on the set of differentially expressed genes identified by edgeR, DESeq2, limma-voom, ELMSeq and rSeqRobust

We further assess the proposed method on a real RNA-seq dataset from The Cancer Genome Atlas (TCGA) project [17], which contains 20,531 genes from 187 prostate adenocarcinoma patient samples. The dataset was downloaded from the TCGA data portal (https://portal.gdc.cancer.gov). In this experiment, we aim at identifying genes associated with pre-operative prostate-specific antigen (PSA), which is an important biomarker for prostate cancer. The data are pre-processed using the procedures described in [11]. We use the Bonferroni correction and determined DE genes using a *p*-value threshold of 0.05/*m*. Figure 1 shows the Venn diagram based on the sets of differentially expressed genes discovered by five methods.

There are twelve genes that are detected by rSeqRobust and ELMSeq, but not by edgeR, DESeq2 and limma-voom: *EPHA5*, *RNF126P1*, *BCL11A*, *RIC3*, *AJAP1*, *CDH3*, *WIT1*, *PRSS16*, *CEACAM1*, *DCHS2*, *CRHR1* and *SRD5A2*. For the majority of these twelve genes, there are existing publications reporting their associations with prostate cancer. For instance, *EPHA5* is known to be underexpressed in prostate cancer [18]. *CEACAM1* is known to suppress prostate cancer cell proliferation when overexpressed [19]. Two of the twelve genes, *CRHR1* and *SRD5A2*, are identified only by rSeqRobust, where *SRD5A2* is associated with racial/ethnic disparity in prostate cancer risk [20].

There are twelve genes that are detected by all five methods: *KANK4*, *RHOU*, *TPT1*, *SH2D3A*, *EEF1A1P9*, *ZCWPW1*, *ZNF454*, *RACGAP1*, *PTPLA*, *POC1A*, *AURKA* and *TIMM17A*. Similarly, there are existing publications reporting their associations with prostate cancer. For instance, RHOU is associated with the invasion, proliferation and motility of prostate cancer cells [21].

## Conclusion & discussion

In this paper, we present a unified statistical model for joint normalization and differential expression detection in RNA-seq. Different from existing methods, we explicitly model sample-specific normalization factors as unknown parameters, so that they can be estimated simultaneously together with detection of differentially expressed genes. Using an *ℓ*_0_-regularized regression approach, our method is robust against large proportion of DE genes and asymmetric DE pattern, and is shown in empirical studies to be more accurate in detecting differential gene expression patterns.

This model generalizes [10] from categorical experimental conditions using an ANOVA-type model to continuous covariates using a regression model. In addition, two hypothesis tests are formulated: i) Is the expression level of a gene associated with any covariates of the regression model? This is the test considered in [10]; ii) Is the expression level of a gene associated with a specific covariate of our interest, when all other variables in the regression model are adjusted for? Although the model is high-dimensional, non-differentiable and non-convex due to the *ℓ*_0_ penalty, we manage to develop an efficient algorithms to find their its solution by making use of the optimality conditions of the *ℓ*_0_-regularized regression. It can be shown that for categorical experimental data, the developed algorithm for the first hypothesis test for the slopes in a regression model with *p* binary covariates reduces to that in [10] for the (*p*+1)-group model.

## Data Availability

The computer programs for the algorithms described in this paper are available at http://www-personal.umich.edu/~jianghui/rseqrobust.

## Abbreviations

ANOVA: Analysis of variance
AUC: Area under the ROC curve
CPM/RPM: Counts/reads per million
DE: Differential expression
LN: Log-normal
NB: Negative binomial
PSA: prostate-specific antigen
RNA-seq: RNA sequencing
ROC: Receiver operating characteristic
RPKM/FPKM: Reads/fragments per kilobase of exon per million mapped reads
TCGA: The Cancer Genome Atlas
TPM: Transcripts per million
